# Notoginsenoside R1 attenuates tendinopathy through inhibiting inflammation and matrix metalloproteinases expression

**DOI:** 10.3389/fphar.2025.1623455

**Published:** 2025-07-08

**Authors:** Qingxin Han, Junying Wu, Yan Li, Yi Tong, Xiaohua Liu, Xiaoqing Hu, Lei Zhang

**Affiliations:** ^1^ Department of Sports Medicine, Wangjing Hospital of China Academy of Chinese Medical Sciences, Beijing, China; ^2^ Department of Sports Medicine, Peking University Third Hospital, Beijing, China

**Keywords:** Notoginsenoside R1, inflammation, matrix metalloproteinases, tendinopathy, experimental validation

## Abstract

**Research purpose:**

The purpose of this study is to demonstrate the effectiveness of Notoginsenoside R1 (NGR1) in treating tendinopathy and to reveal its potential mechanisms.

**Materials and methods:**

This study performed a preliminary network-based assessment of the potential targets that NGR1-associated in the treatment of tendinopathy, which includes PPI network analysis, GO enrichment, KEGG pathway enrichment analysis, and molecular docking. The therapeutic efficacy of NGR1 *in vivo* was then assessed using a collagenase-induced rat model of tendinopathy. Furthermore, the underlying mechanism was explored through LPS-induced inflammatory responses in tenocytes *in vitro.*

**Results:**

Network-based assessment indicated that key targets associated with NGR1 in treating tendinopathy may potentially include IL-6, TNF, and MMP9. *In vivo* studies revealed that NGR1 mitigates the pathological response of tendinopathy induced by collagenase, exhibiting a dose-dependent efficacy, with the 8 μM concentration yielding the most favorable outcomes. RNA sequencing analyses of tenocytes indicated that NGR1 potentially treats tendinopathy by modulating the synthesis of collagen and matrix metalloproteinases, as well as attenuating LPS-induced inflammatory responses. These findings aligned with results obtained from quantitative PCR, ELISA and Western blot analyses.

**Conclusion:**

NGR1 effectively moderates the progression of tendinopathy by modulating inflammatory reactions and matrix metabolism. This discovery offers a promising approach for clinical management of tendinopathy.

## Introduction

Tendinopathy is a relatively complex medical condition that involves a series of lesions in the tendon. The lesion site may exhibit symptoms such as pain, dysfunction, and decreased exercise tolerance (Millar et al., 2021). The lifetime prevalence of tendinopathy in the general population is reported to be 5.9%, while the probability of tendinopathy in the lower extremity is 1%–2%. In comparison, the prevalence of tendinopathy in athletes reaches up to 23.9% (Kujala et al., 2005). Mechanical overuse and the poor healing of acute injury are considered as the two major pathogenic factors of tendinopathy. Hence, tendinopathy is ubiquitous in the sport exposed to high-intensity and repetitive movements, such as running, volleyball, and tennis (Francis et al., 2019; Zwerver et al., 2011), which is also frequent in workers exposed to monotonous repetitive work tasks (Hopkins et al., 2016). Meanwhile, tendinopathy is associated with a few medical diseases such as hypercholesterolemia, rheumatic diseases and diabetes mellitus (Jong et al., 2024; Ahmed, 2016; Soslowsky and Fryhofer, 2016). Other intrinsic risk factors are age, sex, and genetics (Steinmann et al., 2020).

Over the past several decades, the clinical definition and treatment of tendinopathy have been subjects of considerable debate due to insufficient understanding of its pathophysiology. The condition has also been variably described as tendinitis, tenosynovitis, and tendinosis (Millar et al., 2021). Initially regarded as a degenerative disease (Riley, 2008), further pathological investigations into clinical samples of tendinopathy have revealed a significant role for inflammatory responses and immune system activities in its progression (Zhu et al., 2023). Emerging research indicates that in the early stages of healing, a range of inflammatory cytokines and chemokines critically influence the metabolism of tenocytes and extracellular matrix (ECM) (September et al., 2011). This interaction compromises tendon integrity, promotes degeneration, and leads to the formation of fibrovascular scar tissue, which can culminate in tendon tears or ruptures. Building on this insight, targets and signaling pathways involved in inflammatory responses could represent a promising approach to treating tendinopathy. Corticosteroid injections have traditionally been used for this purpose, but their efficacy in controlling inflammation and facilitating tendon repair remains controversial (Blanco et al., 2005; Ko et al., 2022). Research indicates that these injections may impair tendon cell metabolism, resulting in reduced elasticity and tensile strength of the tendon, which in turn increases the risk of tendon rupture (Coombes et al., 2013; Dean et al., 2014). Consequently, the development of new non-corticosteroid anti-inflammatory drugs is vital for the effective treatment of tendinopathy.

**FIGURE 1:**
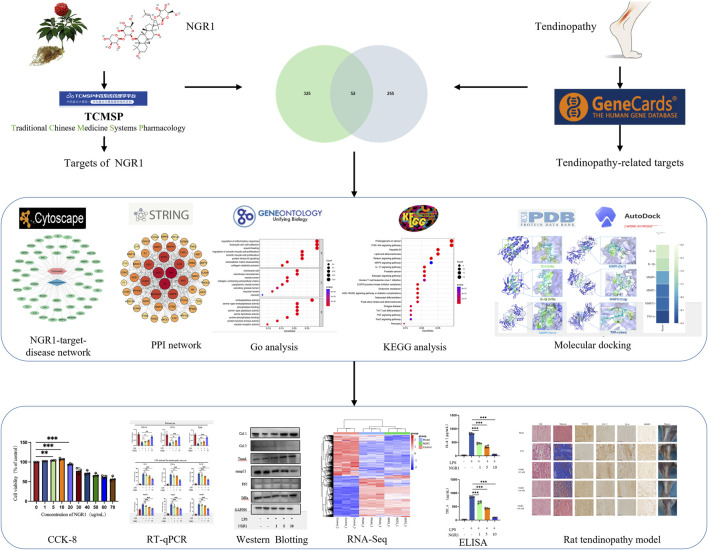
Schematic diagram of the database.

An optimal therapeutic strategy for tendinopathy should not only mitigate inflammation but also enhance the metabolic processes of tendon cells and their matrix, facilitating the recovery of the tendon’s normal structure and function. *Panax notoginseng*, a famous herb in traditional Chinese medicine, is the dried roots and rhizomes of a species in the Araliaceae family. It contains notoginsenosides, ginsenosides, and chikusetsusaponins, which are tetracyclic triterpenoids and are celebrated for their trauma-healing properties in traditional Chinese medicine practices. Contemporary research highlights its anti-inflammatory, antioxidant, anti-apoptotic, and immunomodulatory properties. Laboratory studies have indicated that *P. notoginseng* can modulate collagen metabolism in cells. Given our understanding of its pharmacological properties and the pathophysiology of tendinopathy, *P. notoginseng* and its extracts emerge as promising candidates for tendinopathy treatment. Notoginsenoside R1 (NGR1, Figure 1), a key component of *P. notoginseng*, is effective in mitigating myocardial cell damage caused by reperfusion following a myocardial infarction by modulating signaling pathways associated with oxidative and endoplasmic reticulum stress (Han et al., 2022). Additionally, NGR1 inhibits the activation of the MAPK signaling pathway, which helps reduce liver fibrosis triggered by carbon tetrachloride (Yu et al., 2016). In hyperglycemic conditions, NGR1 also curtails the expression of VEGFA and FGF1, thus decelerating the apoptosis in glomerular podocytes (Gong et al., 2022; Li et al., 2023). Yet, its application in tendinopathy treatment remains unexplored.

Thus, this study employs network analysis to explore the potential targets and signaling pathways for NGR1 in the treatment of tendinopathy. The therapeutic efficacy and potential mechanism of NGR1 to tendinopathy are further validated through experiments using a collagenase-induced rat model of Achilles tendinopathy and cellular molecular biology techniques. This research underscores the therapeutic potential and future applicability of NGR1 in managing tendinopathy (Figure 1).

## Materials and methods

### Network-based analysis and molecular docking

The molecular structure of NGR1 was confirmed through the PubChem database (https://pubchem.ncbi.nlm.nih.gov/). Next, the potential targets of NGR1 were predicted using the PharmMapper database (https://www.lilab-ecust.cn/pharmmapper/), and all target names were verified with the UniProt database (https://www.uniprot.org/). In the GeneCards database (https://www.genecards.org/), the DisGeNET database (https://www.disgenet.org/search/), and the OMIM database (https://omim.org/), “tendinopathy” was used as a keyword to search, download, and integrate all related genes. The intersection between the NGR1 targets and tendinopathy-related genes was used to identify therapeutic targets for NGR1 using the search term “Venn”. A network diagram showing the relationship among NGR1, tendinopathy, and the therapeutic targets was constructed using Cytoscape 3.2.1 software. Then, the topological parameters, including centrality and node degree of the network, were calculated using the network analysis tool to evaluate the significance of the nodes. The protein–protein interaction (PPI) networks of the therapeutic targets were constructed using the STRING database (https://string-db.org/) with the following conditions: “minimum required interaction score = 0.7” and “hide disconnected nodes in the network.” The DAVID v6.8 database (https://david.ncifcrf.gov/) was used to perform Gene Ontology (GO) and Kyoto Encyclopedia of Genes and Genomes (KEGG) pathway enrichment analyses.

Key targets were screened based on three parameters (betweenness, closeness, and degree) in the PPI network of therapeutic targets. Subsequently, molecular docking was performed between NGR1 and the top 5 potential target proteins using AutoDock Vina software. The 3D structures of the target proteins were downloaded from the PDB database (https://www.rcsb.org), and the. mol2 format files of NGR1 were obtained from the TCMSP database. Criteria for selecting target protein crystals included: (1) 3D protein structure determined by X-ray crystallography; (2) crystal resolution less than 2 Å; and (3) reliable genotype protein analysis. Water molecules and original ligands were removed from the target protein using PyMOL. Then, the target protein was imported into AutoDock Tools v1.5.6 for hydrogenation, charge calculation, and combination with non-polar hydrogens, and the result was stored in. pdbqt format. The size of the Grid Box was set to cover the binding area of the target protein and NGR1. Finally, AutoDock Vina was run using CMD command characters for molecular docking, and PyMOL was used to visualize the results.

### Animal model and treatment

8-week-old male Sprague–Dawley (SD) rats were purchased from Beijing Longan Laboratory Animal Breeding Center (Ethics Code. BJLongan-2025–0003, Jan. 16, 2025.). The housing conditions for rats are maintained in an SPF environment. The ambient temperature is maintained at 18°C–22°C, the relative humidity is maintained at 50%–60%, and the average lighting time is 10–14 h. Subsequently, the animals were randomly divided into 5 groups (n = 6, each), including Sham, collagenase-induced tendinopathy (CIT), CIT + NGR1 (1, 4, 8 μM) groups. The tendinopathy model was established following the procedure outlined in the pre-experiment (Supplementary Figure S1) and previous study (Liu et al., 2021). Briefly, following the successful administration of anesthesia, 50 μL type I collagenase (40 mg/mL) was administered via injection into the right Achilles tendon of the rat. The Sham group received equivalent saline injections instead of type I collagenase. After 1 weeks, all groups were treated differently. The rats were locally injected with a therapeutic drug or saline one time per week, depending on their group assignment (Sham: 50 μL saline; CIT: 50 μL saline; CIT + NGR1: 1, 4, 8 μM NGR1 dissolved in saline 50 μL). After 5 weeks, all animals were euthanized, and the right Achilles tendons were collected.

### Histology and immunohistochemistry (IHC) assessment

Rat tendon samples were fixed in 4% neutral-buffered paraformaldehyde (PFA; Solarbio, Beijing, China), embedded in paraffin, and sectioned continuously (5 μm thick). Hematoxylin and eosin (HE) as well as Masson staining were performed according to the protocols. Histological scoring followed the criteria developed by Stoll et al. (2011), where the intact group was assigned a score of 20 points. For immunohistochemical staining (IHC), the paraffin-embedded sections were incubated with 3% H_2_O_2_ for 15 min to inhibit endogenous peroxidase, followed by incubation with 10% goat serum for 1 h at 21 °C to block non-specific antigens. Next, the sections were incubated with specific primary antibodies against collagen I (Col1, ab270993; Abcam, CA, United States; 1:100), collagen III (Col3, ab7778; Abcam; 1:100), IL-6 (ab9324; Abcam; 1:100), matrix metalloproteinase 3 (MMP3, ab52915; Abcam; 1:100) overnight at 4 °C. Subsequently, the sections were incubated for 1 h at 21°C with horseradish peroxidase-conjugated secondary antibodies (PV-6001 and PV-6002, ZSGB-BIO). The integrated OD value of positive staining was evaluated using ImageJ software (National Institutes of Health, MD, United States).

### Primary rat tenocyte isolation and culture

Primary rat tenocytes were isolated from tendon fragments dissected from the Achilles’s tendon of 6-week-old Sprague–Dawley (SD) rat. The rat tendon fragments were mechanically sliced into 2–5 mm^3^ pieces and enzymatically digested with 1% type I collagenase (C0130; Sigma-Aldrich, MO, United States) at 37°C on a shaking incubator for 1 h. After digestion, the cells were resuspended in Dulbecco’s modified Eagle’s medium (DMEM; Gibco) with 20% (v/v) fetal bovine serum (FBS; HyClone, Logan, UT, United States) containing 1 g/L penicillin–streptomycin (Invitrogen, CA, United States) at 37°C in a humid environment with 5% CO_2_. Starting with the initial medium exchange, the concentration of FBS was reduced to 10%, with subsequent changes of the medium occurring bi-daily. Upon achieving a confluence of 85%–90%, the cells should be passaged. Tenocytes at passage three (P3) are designated for use in various experimental assays.

### Cell viability assay

Cell proliferation and viability were assessed using the Cell Counting Kit-8 (CCK-8). Tenocytes were initially seeded in 96-well plates at a density of 1 × 10^3^/well. Subsequently, various concentrations of NGR1 (Purity: HPLC ≥98%; CAS No.: 80,418–24–2; Sichuan Weikeqi Biotechnology Co., Ltd., China.) were introduced after tenocyte adhesion and incubated for 24 h. Afterward, CCK-8 reagent (10 μL/well) was added and allowed to incubate for 2 h. Use a microplate reader to measure the absorbance at 450 nm wavelength to assess cell viability.

Cell viability is calculated by optical density (OD) using the formula:

$$\frac{{\bar{OD}}_{\text{experimental}}-{\bar{OD}}_{\text{blank}}}{{\bar{OD}}_{\text{control}}-{\bar{OD}}_{\text{blank}}}\times 100\%$$

### RNA extraction and real-time qPCR

Tenocytes were starved for 6 h without FBS and subsequently treated with LPS (250 ng/mL; L2880-10 MG; Sigma-Aldrich, MO, United States) and NGR1 (1, 5, 10 μg/mL) for 24 h. Total RNA was extracted from primary cultured tenocytes using the RNeasy Plus Mini Kit (Cat. No. 74136, QIAGEN). Purified RNA (2 μg) was reverse-transcribed using the RevertAid First Strand cDNA Synthesis Kit (Thermo Fisher Scientific, Boston, MA, United States). The real-time qPCR was performed on the Applied Biosystems StepOnePlus Real-Time PCR System (Foster City, CA, United States) using SYBR Green PCR Master Mix (Toyobo, Japan). The expression levels of target mRNA were normalized to those of glyceraldehyde-3-phosphate dehydrogenase (GAPDH) RNA. Relative gene expression was calculated using the 2^−ΔΔCT^ method and expressed as fold change. Each real-time qPCR assay was performed in triplicate, with at least three different biological replicates. Primer sequences are listed in Supplementary Table S1.

### Immunofluorescence analysis

The tenocytes were first rinsed with PBS, then fixed with 10% neutral-buffered formalin at 21°C for 30 min. The cells were treated with Triton X-100 (Beyotime Biotechnology, Beijing) for 10 min to penetrate the cell membrane, followed by blocking with goat serum (Beyotime Biotechnology) for 1 h to prevent nonspecific binding. The cultured cells were incubated overnight at 4°C with primary antibodies against Col1 (ab270993; Abcam; 1:100). Subsequently, the cells were washed three times with PBS and incubated at 21°C for 1 h with Alexa Fluor 488-conjugated goat anti-rabbit secondary antibodies (A-11008, Thermo Fisher Scientific; 1:200). Nuclei were stained with DAPI (Beyotime Biotechnology, Jiangsu) for 10 min. Finally, the samples were rinsed with PBS and observed under a confocal microscope (Olympus Life Science, Tokyo, Japan).

### ELISA assay

IL-6 and TNF-α production in the supernatants was quantified by ELISA kits according to the manufacturer’s protocol. The IL-6 and TNF-α ELISA kits (PI328 and PT516) were from Beyotime Biotechnology (Jiangsu, China).

### Protein extraction and Western blot analysis

Tenocytes were starved for 6 h without FBS and subsequently treated with LPS (250 ng/mL) and NGR1 (1, 5, 10 μg/mL) for 4 days. Then, tenocytes were lysed in radioimmunoprecipitation assay (RIPA) lysis buffer containing protease inhibitors and/or a phosphatase inhibitor cocktail. The protein concentration was determined using the Bradford Protein Assay Kit (Beyotime, China). The total cell lysates were prepared in lysis buffer (150 mM NaCl, 1% Nonidet P-40, 50 mM Tris, 5 mM NaF), separated by SDS polyacrylamide gel electrophoresis (PAGE), and transferred to a polyvinylidene fluoride (PVDF) membrane. After blocking with 5% BSA in 0.1% Tween 20 TBS (TBST), the membranes were incubated with the corresponding primary antibodies overnight at 4 °C. After washing three times with TBST, the membranes were incubated with secondary antibodies at 21°C for 1 h and visualized using the BIORAD ChemiDoc XRS + system.

Proteins were analyzed using antibodies against Col 1 (ab270993; Abcam, CA, United States; 1:1,000), Col 3 (ab7778; Abcam; 1:1,000), tenomodulin (Tnmd, ab203676; Abcam; 1:1,000), P65 (ab32536; Abcam; 1:1,000), IκBα (ab32518; Abcam; 1:1,000), GAPDH (4,970, Cell Signaling Technology). Anti-mouse (ZB-2305, HRP-conjugated) and antirabbit (ZB-2301, HRP-conjugated) secondary antibodies were purchased from ZSGB-BIO (Beijing, China; 1:1,000).

### RNA sequencing (RNA-seq) for the tenocyte transcriptome

We performed RNAseq analysis on rat tenocytes using the NovelBrain Cloud Analysis Platform. In the experimental setup, tenocytes in the control group remained untreated. The model group received treatment with LPS, while the NGR1 group was treated with a combination of LPS and NGR1 (10 μg/mL). After a 2-day treatment, total RNA was extracted from the rat tenocytes using TRIzol reagent. cDNA libraries were constructed for each pooled RNA sample (per rat) using the VAHTS™ Total RNA-seq (H/M/R) Kit. Differential gene and transcript expression analyses were performed using TopHat and Cufflinks, while HTseq was used to count the gene and lncRNA counts. The FPKM method was employed to determine gene expression levels. We applied the DESeq algorithm to identify differentially expressed genes (DEGs). Significant analysis was performed using P values and false discovery rate (FDR) analysis. DEGs were considered significant if they had an $\left|\log2\text{FoldChange}\right|>1$ and an FDR <0.05. GO analysis was performed to elucidate the biological implications of the differentially expressed genes, encompassing biological processes (BP), cellular components (CC), and molecular functions (MF). GO annotations were downloaded from NCBI (http://www.ncbi.nlm.nih.gov/), UniProt (http://www.uniprot.org/), and Gene Ontology (http://www.geneontology.org/). Pathway analysis was conducted to identify significantly influenced pathways in which the DEGs were involved, according to the KEGG database. Fisher’s exact test was used to identify significantly influenced GO categories and pathways. The threshold of significance was defined by the *p* value.

### Hot plate test

The pain response in experimental animals was evaluated using a hot plate test. Briefly, the animals were placed on a hot plate analgesia meter (Ugo Basile, Italy) maintained at 55°C. The latency from initial hind paw contact to the onset of nociceptive behaviors (e.g., paw shaking, jumping, or licking) was recorded. Each animal underwent three trials at 15-min intervals, and the mean response latency was calculated as the final pain threshold. All behavioral assessments were performed by observers blinded to experimental group assignments.

### Statistical analysis

Data were analyzed using GraphPad Prism 8.0.1 (244) software (San Diego, United States). Differences between multiple groups were assessed using ANOVA with Tukey’s *post hoc* test, and differences between two groups were assessed using an unpaired *t*-test. All data are presented as means ± SD, and statistical significance was established at *p* < 0.05 (*), *p* < 0.01 (**), and *p* < 0.001 (***). All the statistical analysis was independently conducted by two authors (J. W. and Y. L.) whom were blinded with the experimental protocol under the consideration of data reliability.

## Results

### Targets investigation of NGR1 on tendinopathy

**FIGURE 2:**
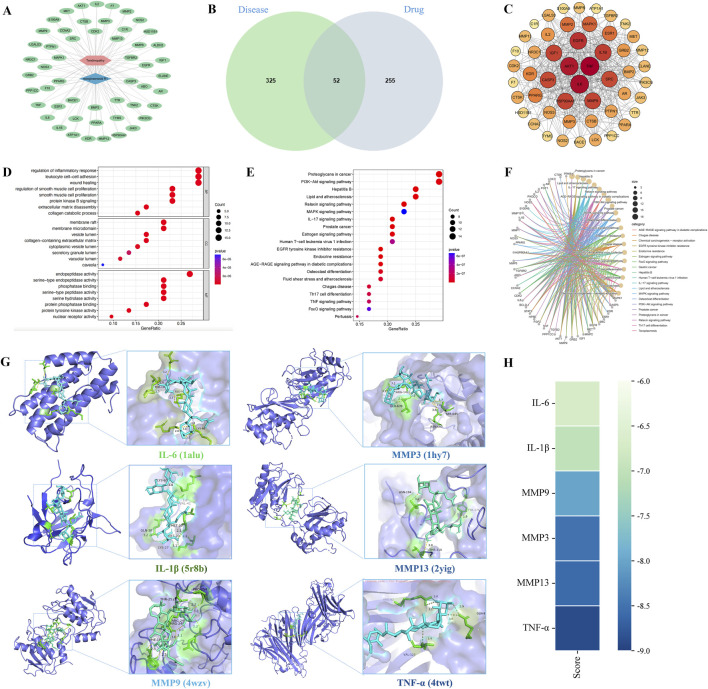
Mechanisms underlying the effects of NGR1 on tendinopathy. **(A)** In the drug-disease-target network, the blue diamond symbolizes NGR1, the pink hexagon denotes tendinopathy, and the green ovals represent the targets. **(B)** The Venn diagram illustrates the overlap between 325 disease-associated targets and 255 drug-related targets, identifying 52 shared targets. **(C)** The PPI network diagram highlights nodes with higher connectivity in red, indicating their central role. **(D)** GO analysis was conducted to analyze the common biological functions of the 52 targets selected from the cellular component (CC), molecular function (MF), and biological process (BP). **(E)** KEGG pathway enrichment analysis underscores critical pathways involved in signal transduction and immune responses. **(F)** The core target-pathway relationship network depicts the distribution of targets across various biological pathways. **(G)** Molecular docking analysis demonstrates the interaction modes and binding energies of IL-6, IL-1β, MMP3, MMP13, MMP9, and TNF-α with NGR1, with binding energies below −5 kcal/mol, suggesting stable interactions. **(H)** The binding energy heatmap of NGR1 and all six core targets.

In total, 52 therapeutic targets were obtained through the intersections between NGR1 targets (n = 307) and tendinopathy targets (n = 377) (Figure 2A). Based on these data, the NGR1−tendinopathy− target network was constructed and visualized (Figure 2B). We constructed a PPI network (Figure 2C) to investigate the interactions among the targets, which were determined based on the degree of connectivity of each target in the network. These targets exhibit a high degree of interaction within the PPI network, including tumor necrosis factor (TNF, degree = 43), interleukin-6 (IL-6, degree = 42), serine/threonine kinase 1 (ATK1, degree = 40), endothelial growth factor receptor (EGFR, degree = 36), interleukin-1B (IL-1B, degree = 34), and Matrix metalloproteinase 9 (MMP9, degree = 33) (Supplementary Table S2). GO enrichment analysis was conducted to evaluate the common biological functions of the 52 targets (Figure 2D). KEGG enrichment analysis showed 147 related pathways with a significance threshold of *p* < 0.05. Among them, the PI3K/AKT signaling pathway and proteoglycans in cancer showed the highest correlation (Figure 2E). Concurrently, we constructed a core target-pathway relationship network to elucidate gene functions across distinct signaling pathways (Figure 2F).

Following the analysis of PPI data, six key targets (IL-6, IL-1β, MMP3, MMP13, MMP9, and TNF-α) were selected for further investigation. Subsequent molecular docking with the ligand NGR1 revealed significant interactions, as detailed in Figure 2G, which illustrates the three-dimensional structures of the proteins and the specific interactions at their binding sites. The binding energies recorded for all interactions were below −5 kcal/mol, suggesting robust and stable docking configurations (Figure 2H).

### NGR1 effectively alleviates the pathological process of tendinopathy in rat

**FIGURE 3:**
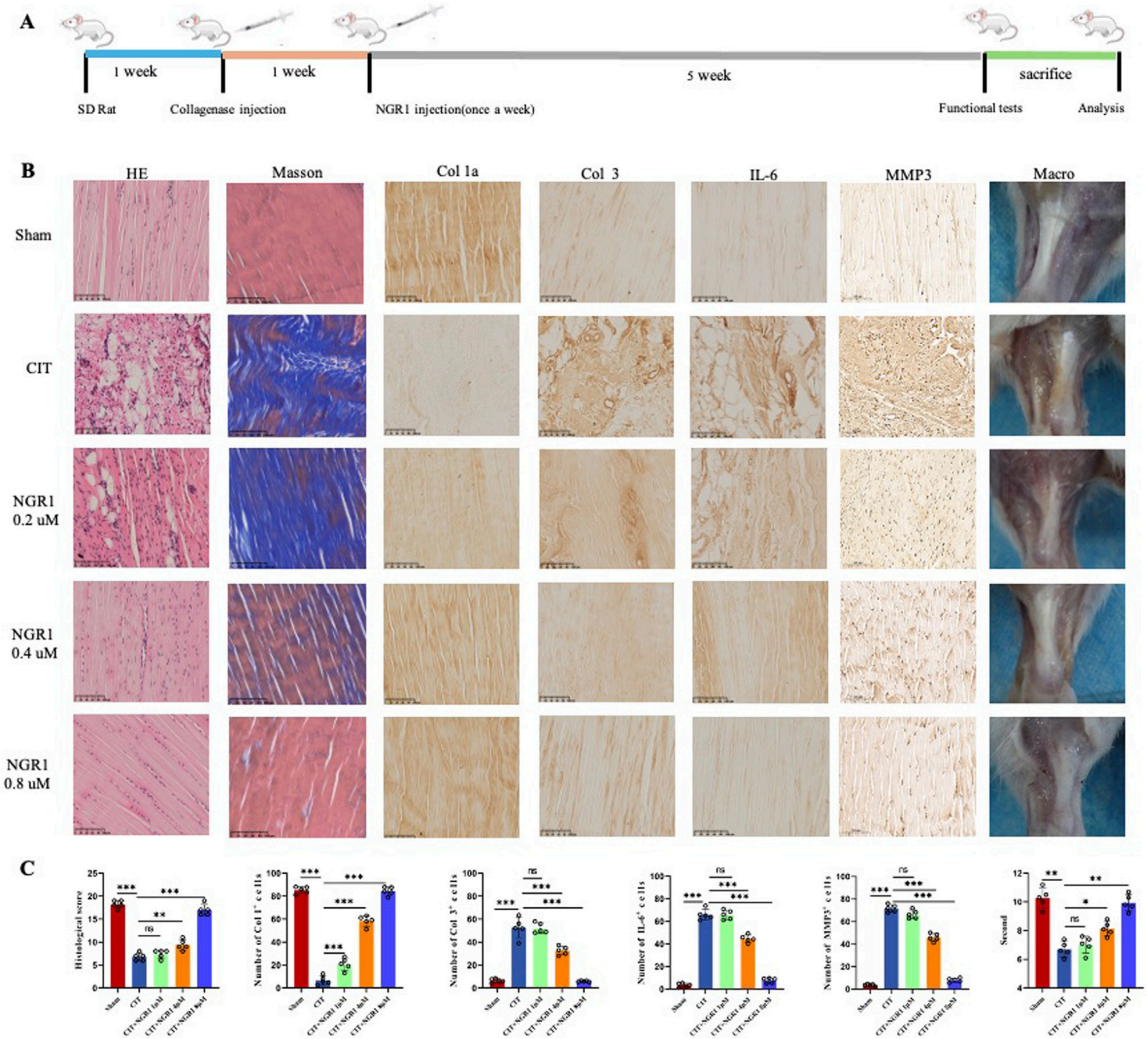
NGR1 alleviated tendinopathy. **(A)** The process of treating tendinopathy with NGR1. **(B)** HE and Masson staining of tendon, immunohistochemistry of Col 1a, Col 3, IL-6 and MMP3 in the five groups (sham, CIT, CIT + NGR1 (1, 4, 8 μM) groups), and corresponeded macro-picture; all the scale bars = 100 μm. **(C)** Histological score, quantitative analysis of immunohistochemical staining of the Col 1, Col 3, IL-6 and MMP3 expression *in vivo*, and pain response times of rats in each group (n = 5). The black arrowheads indicate pathological manifestations including lipid vacuoles, inflammatory cell infiltration, and ectopic ossification.

**FIGURE 4:**
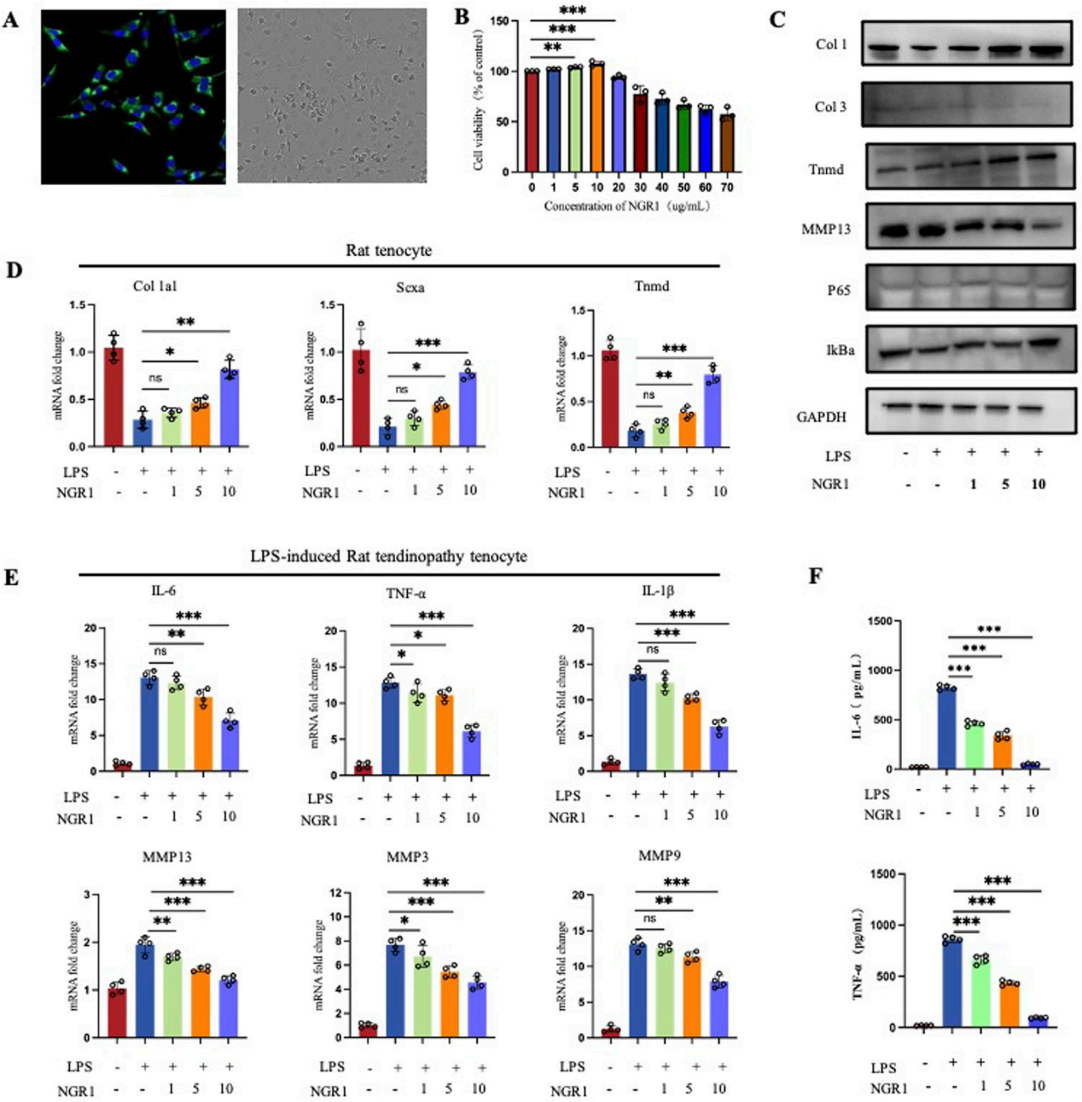
**(A)** Immunofluorescence staining to detect the expression of the tenocyte marker Col 1 and observation of cell morphology under light microscope. **(B)** CCK-8 Assays of cell viability of tenocytes treated with varying concentrations of NGR1. **(C)** Western blot result of tendinopathy-related gene expression in tenocytes (Col 1, Col 3, Tnmd, and MMP13) and NF-κB pathway-related proteins (IκBα and p65) in LPS-induced tenocytes treated with varying concentrations of NGR1 (1, 5, 10 ug/mL). **(D)** Quantification of Col 1a1, Scxa, Tnmd mRNA (n = 4). **(E)** Quantification of IL-6, TNF-α, IL-1β, MMP3, MMP9, and MMP13 (n = 4). **(F)** ELISA detection of IL-6 and TNF-α expression levels.

The healing process of tendinopathy was evaluated by morphological analysis including macro view, HE and Masson staining, and IHC. Macroscopically, it is evident that the CIT group is characterized by a substantial amounts of inflammatory hyperplasia (pale and deep yellow tissues) covering the tendon structures. Meanwhile, HE and Masson staining of tendons revealed that the collagen fibers in the healthy Achilles tendon tissue of the sham group were orderly arranged. In contrast, the control group’s Achilles tendon tissue exhibited typical tendinopathy pathological features, such as disorganized arrangement of collagen bundles, fragmentation of collagen fibers, fat infiltration and the ingrowth of neovessels, which was observed a gradation of therapeutic reversal in the low, medium, and high dosage groups of NGR1. IHC staining revealed that NGR1 exhibited a dose-dependent therapeutic effect on tendinopathy, including reducing the expression of the inflammatory cytokine IL-6, downregulating Col3 and MMP3 expression, and upregulating Col1 expression at 5 weeks (Figure 3). The high-dose (8 μM) NGR1 group exhibited the best therapeutic effects. This indicates that NGR1 plays a therapeutic role in the early inflammatory stage of tendinopathy with 5 weeks. In addition, the hot plate test showed that the pain response of rat in the CIT group was more sensitive than other group after treatment (Figure 4C).

Histological staining results showed that local administration of NGR1 has no negative impact on healthy tendon tissue (Supplementary Figure S2A). Additionally, evaluation of heart, liver and kidney samples showed no significant differences compared with the control group, confirming the absence of systemic side effects (Supplementary Figure S2B–D). Consequently, NGR1 emerges as a safe therapeutic agent capable of effectively alleviating tendinopathy progression in rat via local administration.

### NGR1 reduced the expression of inflammatory cytokines and MMPs proteins *in vitro*

The tenocytes were first identified. Immunofluorescence results showed high expression of Col 1 in the cultured cells, and light microscopy revealed the characteristic spindle-shaped morphology, consistent with tenocyte features (Figure 4A). CCK-8 test results established a safe concentration range for NGR1 (0–10 μg/mL), considered the therapeutic dose of the *in vitro* study (Figure 4B). According to the real-time qPCR results, NGR1 enhanced the expression of Col 1a1, scleraxis (Scx), and Tnmd (Figure 4D), findings that are corroborated by Western blot analysis (Figure 4C). NGR1 effectively suppressed the expression of pro-inflammatory cytokines IL-1β, IL-6, and TNF-α, while also downregulating MMP3, 9, and 13 (Figure 4E). Furthermore, NGR1 reduced the production of IL-6, and TNF-α (Figure 4F) in the supernatants.

**FIGURE 5:**
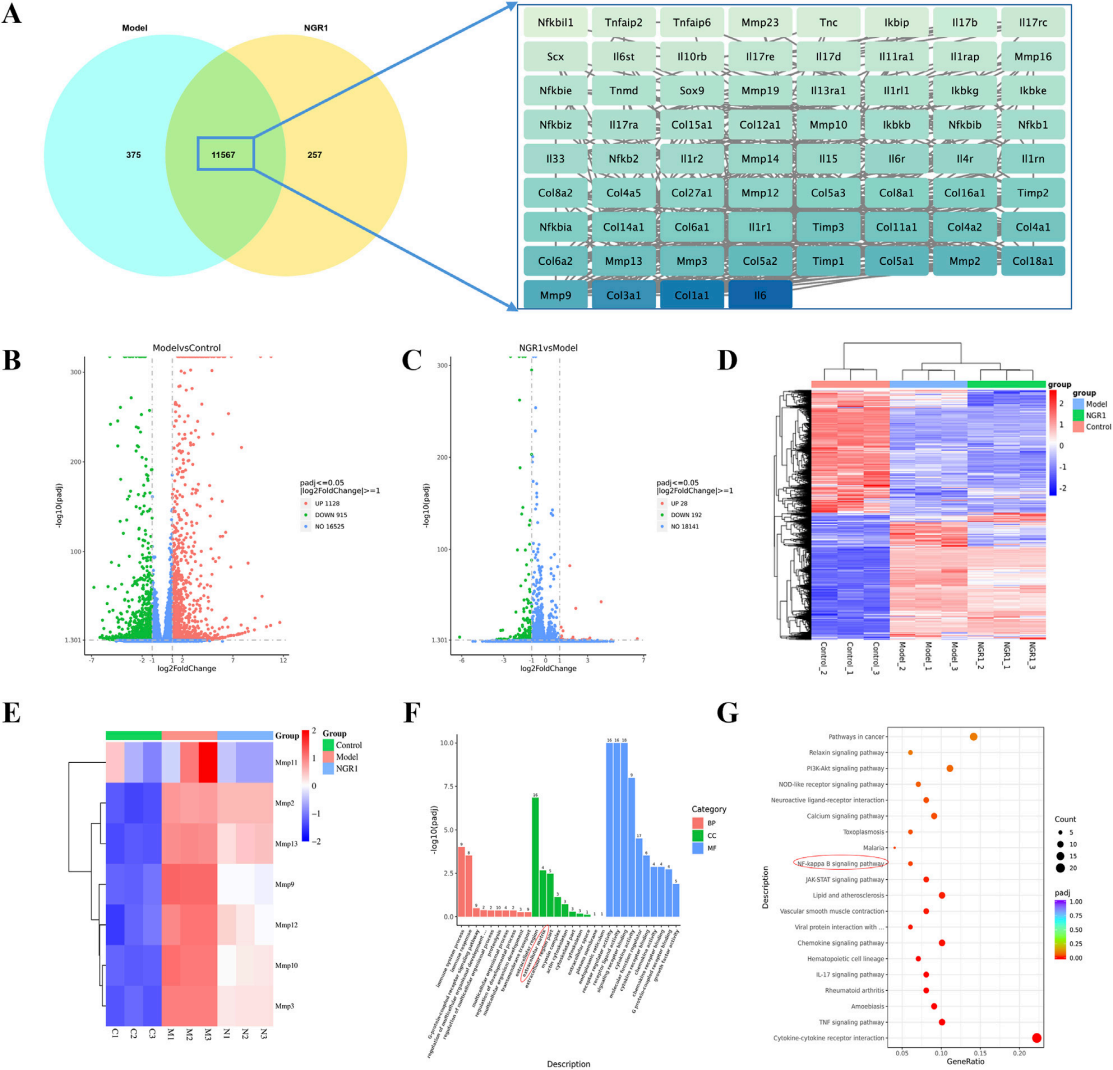
The results of RNA-seq. **(A)** The Venn diagram illustrates the common targets between NGR1 and Model group with core targets. **(B)** Volcano Plot Analysis Between the Model and Control Groups. **(C)** Volcano Plot Analysis Between the Model and NGR1 Groups. **(D)** Heatmap of three groups **(E)** Heatmap of MMPs. **(F)** GO analysis of Between the Model and Control Groups. KEGG analysis of Between the Model and Control Groups. **(G)** KEGG analysis of Between the Model and NGR1 Groups.

RNA sequencing analysis revealed that the Pearson correlation coefficients for samples within each group exceed 0.98, demonstrating a high degree of correlation among them. The analysis also identified 11,567 common targets between the NGR1 and Model group, among which Col 1a1, Col 3a1, and IL-6 were the core genes. (Figure 5A). Differential expression analysis revealed that, in comparison to the control group, the model group exhibits upregulation in 1,128 genes and downregulation in 915 genes. When comparing the NGR1 group to the model group, there are increases in expression levels of 28 genes and decreases in 192 genes (Figures 5B,C). Cluster analysis indicated that expression levels of MMP2, 3, 9, 10, 11, 12, and 13 were elevate in the model group compared to the control group. However, these levels decreased after treatment with NGR1, suggesting that NGR1 may effectively manage tendinopathy by modulating extracellular matrix metabolism (Figures 5D,E). Based on the GO analysis results, the most significant term in the BP category is immune system process; in the CC category, extracellular region shows the highest significance, followed closely by “extracellular space”; in the MF category, signaling receptor activator activity exhibits the highest significance level, followed by molecular transducer activity (Figure 5F). The KEGG analysis results suggested that the signaling pathways potentially involved in the treatment of tendinopathy with NGR1 include the TNF signaling pathway, NF-κB signaling pathway, and PI3K-Akt signaling pathway, among others (Figure 5G).

## Discussion

Tendons represent a highly specialized form of connective tissue, primarily tasked with the transmitting forces generated by muscle contractions to the skeletal system, facilitating movement. During muscle contraction, tendons effectively convey this force to the bones, resulting in joint motion. This critical mechanism of force transmission underscores the essential role of tendons in ensuring both mobility and stability within the human body (Lin et al., 2022). In mature, healthy tendons, the collagen fibers are well-organized, primarily consisting of collagen (specifically Col1), elastin, proteoglycans, and water. Collagen, which is the most abundant protein in tendons, constitutes about 65%–80% of their dry weight, offering essential strength and resilience. Elastin, meanwhile, imparts the necessary elasticity and flexibility to the tendon structure (Millar et al., 2021). Tendinopathy involves structural derangements, including collagen disorganization, hypervascularization, ectopic ossification, and a reduced Col1/Col3 ratio. Tendinopathy represents a degenerative condition that progresses through three interrelated phases: inflammation, proliferation, and remodeling. The HE staining and IHC findings from our model group align with the known pathological characteristics of tendinopathy. This confirms the successful establishment of a tendinopathy model, enhancing the reliability of the therapeutic outcomes observed with NGR1 treatment.

In this study, we first utilized network-based analysis to assess potential targets for NGR1 in treating tendinopathy. By intersecting the targets of NGR1 with those associated with tendinopathy, we constructed a PPI network, and the results showed that targets such as IL-6, TNF, AKT1, MMP9, and MMP3 are valuable in assessment. Furthermore, the molecular docking results presented the stable and high-affinity binding of NGR1 to TNF-α, IL-6, MMP3, MMP9, and MMP13, indicating that NGR1 may exert its therapeutic effects on tendinopathy by modulating inflammatory responses and cellular and extracellular matrix metabolism. We then conducted *in vitro* experiments for validation. Initially, tendon cells were isolated from rat Achilles tendons, and qPCR and Western blot assays confirmed their primary tendon cell identity by showing expression of Col1, Col3, Tnmd, and Scx genes. Following LPS induction, there was an increase in IL-6, TNF-α, and IL-1β expression in the tendon cells, which decreased upon treatment with NGR1, demonstrating its potent anti-inflammatory properties. Despite longstanding debates regarding the presence of inflammation in the development of tendinopathy, emerging evidence from advanced pathological assessments and genetic studies increasingly supports the significant role of inflammation, particularly in the early stages of the disease. Research indicated that the infiltration of macrophages and the rise in inflammatory cytokines disrupt the normal state of tendons, leading to pathological changes and ectopic ossification. Macrophages, versatile in their function, significantly influence tendinopathy by adopting either M1 or M2 phenotypes, thereby impacting both inflammation and tissue repair (Gracey et al., 2020; Lin et al., 2020). In their study, Tang et al. (2024) analyzed transcriptomic data and clinical features from 126 tendinopathy cases, classifying them into three distinct subtypes: the hypoxic atrophic subtype with a white appearance (Hw) characterized by reduced neovascularization; the inflammatory proliferative subtype with a white appearance (Iw), showing moderate increases in inflammatory markers; and the inflammatory proliferative subtype with a red appearance (Ir), which displays extensive neovascularization and inflammation, associated with severe joint dysfunction. This classification underscores the critical importance of managing inflammation in the therapeutic strategy for tendinopathy. The IHC findings from our study indicate that IL-6 expression levels were elevated in the model group compared to the sham-operated group. Following treatment with NGR1, there was a marked reduction in IL-6 expression, which showed a dose-dependent relationship; the highest dosage yielded the most significant therapeutic outcomes. Furthermore, there was an increase in Col1 levels and changes in Col 3 expression, confirming the effective *in vivo* treatment potential of NGR1.

**FIGURE 6:**
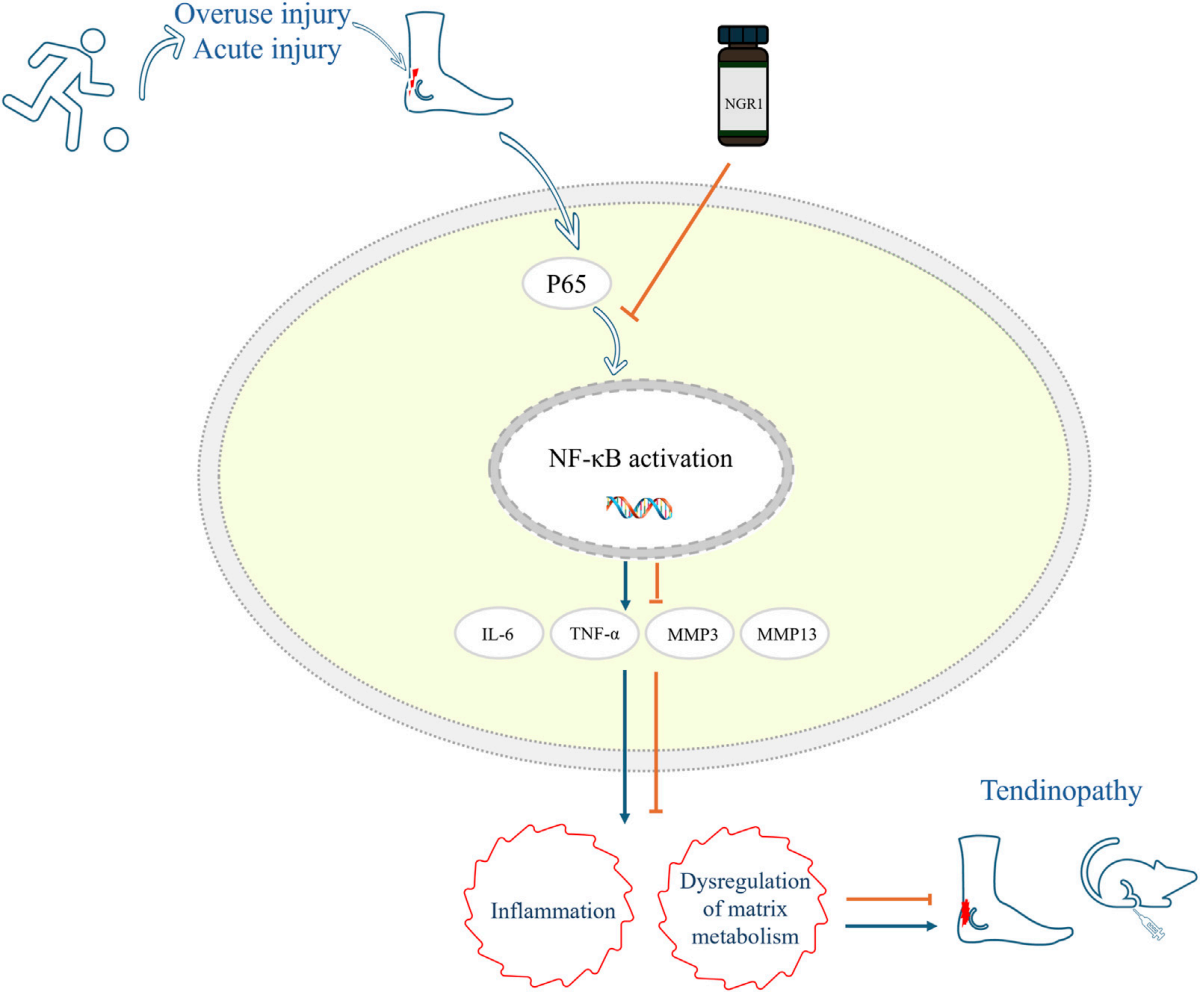
Mechanism diagram of NGR1 in the treatment of tendinopathy in this study. NGR1 Inhibits the Activation of the NF-kB Signaling Pathway and a subsequent reduction in inflammation and regulation of matrix metabolism, thereby contributing to the attenuation of tendinopathy progression.

Moreover, the metabolism of the ECM is crucial in the development and progression of tendinopathy. MMPs are a family of zinc-dependent endopeptidases essential for the degradation of ECM components. They are crucial in maintaining ECM integrity by catalytically breaking down structural proteins such as laminin, fibronectin, and various types of collagens. This enzymatic activity allows MMPs to regulate tissue remodeling and repair. They also play a pivotal role in pathological processes where ECM degradation contributes to disease progression (Somerville et al., 2003). MMP3, also known as stromelysin-1, plays a pivotal role in the ECM remodeling process by stimulating the activity of other MMPs. This stimulates a cascade effect that enhances the breakdown of various ECM proteins. This capability of MMP3 to act as a catalyst for other MMPs underscores its significant influence in both physiological and pathological tissue remodeling (El Khoury et al., 2016). In the context of tendinopathy, MMP9 is particularly important due to its ability to degrade collagen, a primary structural component of tendons. The overexpression of MMP9 can disrupt the normal architecture and mechanical properties of the tendon matrix, contributing to the degeneration of tendon tissues. This enzymatic activity exacerbates the breakdown of collagen fibers, which compromises tendon strength and elasticity, leading to further injury and pain (Sánchez-Sánchez et al., 2020; Tsai et al., 2013). In this study, RNA sequencing results indicated that the expression levels of MMP3, MMP9, and MMP13 in tendon cells were significantly increased following LPS induction. However, introducing NGR1 markedly reduced MMPs expression. Concurrently, Western blotting experiments and IHC analysis of tendon samples demonstrated that NGR1 could enhance the expression of Col1 and Tnmd, while reducing the expression of Col3. The IHC findings from our study indicate that MMP3 expression levels were elevated in the model group compared to the sham-operated group. Following treatment with NGR1, there was a marked reduction in MMP3 expression, which showed a dose-dependent relationship; the highest dosage yielded the most significant therapeutic outcomes. These findings suggest that NGR1 not only treats tendinopathy by modulating inflammatory responses but also potentially by promoting the repair of the ECM (Figure 6).

Although network-based analysis provided a useful foundation for identifying potential molecular targets of NGR1, this approach is inherently predictive and exploratory. The *in silico* results were used solely as a guide for subsequent experimental validation, rather than as standalone evidence. Databases such as GeneCards and DisGeNET are interconnected and may include redundancies, which limits the specificity of the predictions. The experimental validation is critical to confirm computational predictions. While our *in vivo* and *in vitro* experiments support several predicted interactions, further mechanistic studies are needed to confirm direct molecular targets and signaling pathways.

## Conclusion

This research has confirmed that NGR1 mitigates the progression of tendinopathy by suppressing inflammatory responses and modulating ECM metabolism, which enhances tendon healing. Animal studies indicate a dose-dependent efficacy of NGR1, with the highest dose resulting in the most substantial improvement in tendon repair quality. Further evidence from RNA sequencing and Western blot analyses suggests that NGR1’s therapeutic effects may be mediated through its interactions with the NF-κB pathway. Nevertheless, future studies are required to elucidate the specific interactions between NGR1 and the targets within the NF-κB signaling pathway.

## Data Availability

The data presented in the study are deposited in the NCBI repository, accession number PRJNA1283482.
