# Supplementary material for: Notoginsenoside R1 attenuates tendinopathy through inhibiting inflammation and matrix metalloproteinases expression
Source: Front Pharmacol. 2025 Jul 8;16:1623455. doi: 10.3389/fphar.2025.1623455 (PMC12279872; doi:10.3389/fphar.2025.1623455)
Supplement: Supplementary file 1 [file DataSheet1.docx]

| **Gene** | **Sequences** | **Species** |
| --- | --- | --- |
| Col1a1 | Forward: TGGCAACCTCAAGAAGTCCC | Rat |
|  | Reverse: ACAAGGGTGCTGTAGGTGAA |  |
| Tnmd | Forward: CGCCGCACCAGACAAG | Rat |
|  | Reverse: TAAAGGTTCACAGACCCTGC |  |
| Scxa | Forward: AGATGCTCGGTTTC | Rat |
|  | Reverse: CACGGCTATAGAGTTCA |  |
| IL-6 | Forward: AAGAAGCCACCCTCAAGCC | Rat |
|  | Reverse: AGCAAGGACACCCGCACTC |  |
| TNF-a | Forward: TCTTCTGCCTGCTGCACTTC | Rat |
|  | Reverse: CTTGCGGGTTTGCTACTACG |  |
| IL-1β | Forward: CCAGGACATGCTAGGGAGC | Rat |
|  | Reverse: CAGAGGCAGGGAGGGAAA |  |
| Mmp3 | Forward: ACCCACCTTACATACAGGATT | Rat |
|  | Reverse: ACCCACCTTACATACAGGATTG |  |
| Mmp9 | Forward: CCAAGAGTCGCCAGTACCTC | Rat |
|  | Reverse: CCAAGAGTCGCCAGTACCTC |  |
| Mmp13 | Forward: AAGGACCCTGGAGCACTCATGTTT | Rat |
|  | Reverse: TGGCATCAAGGGATAAGGAAGGGT |  |
| GAPDH | Forward: CCTGCACCACCAACTGCTTA | Rat |
|  | Reverse: CATCACGCCACAGCTTTCCA |  |

**Table S1: Primers for real-time RT-PCR used in this study**

**Table S2 The results of PPI network construction**

| **Name** | **Degree** | **BetweennessCentrality** | **ClosenessCentrality** |
| --- | --- | --- | --- |
| TNF | 43 | 0.110256 | 0.890909 |
| IL6 | 42 | 0.101171 | 0.875 |
| AKT1 | 40 | 0.08398 | 0.844828 |
| EGFR | 36 | 0.054591 | 0.790323 |
| IL1B | 34 | 0.034448 | 0.765625 |
| MMP9 | 33 | 0.03272 | 0.753846 |
| HSP90AA1 | 33 | 0.040249 | 0.742424 |
| IGF1 | 33 | 0.030846 | 0.742424 |
| SRC | 32 | 0.031811 | 0.742424 |
| CASP3 | 32 | 0.030671 | 0.742424 |
| MAPK1 | 28 | 0.022439 | 0.690141 |
| ESR1 | 26 | 0.009827 | 0.671233 |
| MMP2 | 26 | 0.011415 | 0.680556 |
| PPARG | 24 | 0.01214 | 0.662162 |
| KDR | 23 | 0.005992 | 0.653333 |
| MMP3 | 20 | 0.005 | 0.628205 |
| IL2 | 20 | 0.003214 | 0.620253 |
| GRB2 | 19 | 0.008827 | 0.6125 |
| CTSB | 18 | 0.007422 | 0.6125 |
| AR | 17 | 0.003914 | 0.597561 |
| PTPN1 | 17 | 0.00173 | 0.597561 |
| NOS3 | 17 | 9.71E-04 | 0.597561 |
| BMP2 | 16 | 0.001508 | 0.590361 |
| NR3C1 | 16 | 0.003007 | 0.590361 |
| MET | 15 | 6.55E-04 | 0.583333 |
| PPARA | 15 | 0.002764 | 0.583333 |
| LCK | 15 | 0.001454 | 0.583333 |
| CTSK | 15 | 0.002255 | 0.583333 |
| CDK2 | 15 | 0.006499 | 0.583333 |
| LGALS3 | 14 | 0.041075 | 0.583333 |
| NOS2 | 13 | 6.46E-04 | 0.569767 |
| TGFBR2 | 12 | 0.002628 | 0.563218 |
| ELANE | 12 | 0.00219 | 0.563218 |
| MMP13 | 12 | 7.53E-04 | 0.563218 |
| JAK3 | 11 | 1.99E-04 | 0.556818 |
| CCNA2 | 11 | 0.001336 | 0.538462 |
| MMP8 | 10 | 3.86E-04 | 0.550562 |
| MMP12 | 9 | 3.10E-04 | 0.532609 |
| PIK3CG | 9 | 1.26E-04 | 0.544444 |
| BACE1 | 9 | 6.54E-05 | 0.544444 |
| HSD11B1 | 6 | 1.34E-04 | 0.5 |
| TTR | 6 | 0.001022 | 0.515789 |
| TNK2 | 6 | 7.73E-05 | 0.505155 |
| PPP1CC | 6 | 3.68E-04 | 0.505155 |
| S100A9 | 5 | 0 | 0.5 |
| TYMS | 5 | 6.07E-05 | 0.49 |
| F7 | 4 | 2.83E-04 | 0.49 |
| ATP1A1 | 4 | 0 | 0.485149 |
| F10 | 3 | 0 | 0.485149 |
| C1R | 1 | 0 | 0.371212 |


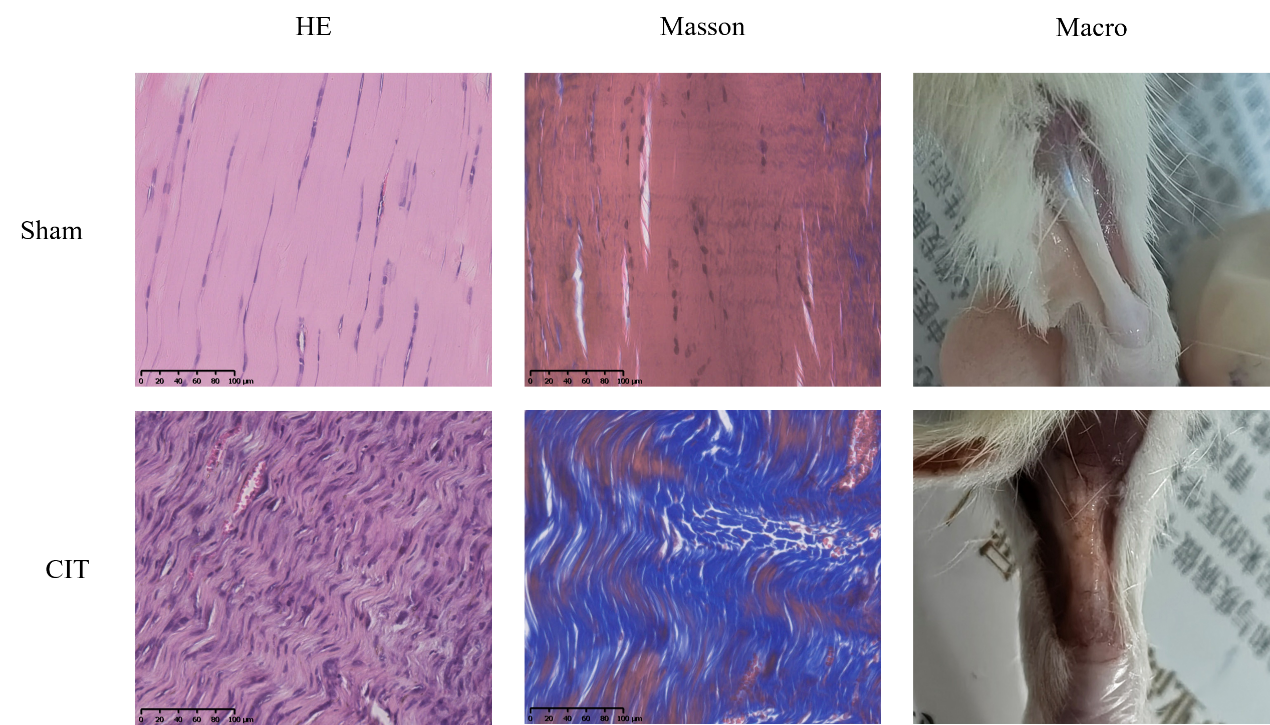


Fig. S1. Construction of a collagenase-induced tendinopathy model in rat. all the scale bars are 100 μm.


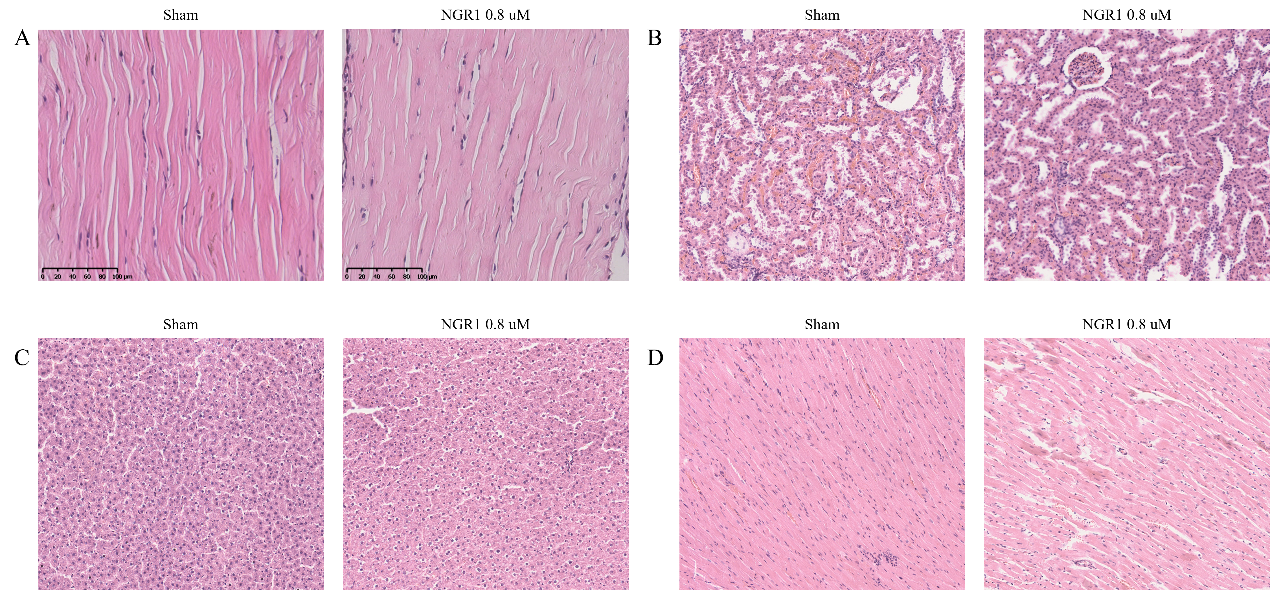


Fig. S2. The biological safety of NGR1. HE stainning results of representative tissue sections of rats (heart, liver, and kidney) after injection of NGR1 (8 μM). Scale bar, 100 μm. (A) Achilles tendon tissue. (B) Heart. (C) Liver. (D) Kidney.
